# Osmium(viii)-catalyzed oxidative degradation of paracetamol by chloramine-T in an aqueous alkaline medium: computational screening and mechanistic pathways

**DOI:** 10.1039/d5ra03805g

**Published:** 2025-09-09

**Authors:** Deepmala Pareek, Asha Rolaniya, Akta Yadav, Nikita, Menka Bhasin, Anita Meena, Mamta Ranka, Priyanka Jain, Riya Sailani

**Affiliations:** a Guru Tegh Bahadur 4th Centenary Engineering College Rajouri Garden New Delhi India; b Department of Chemistry, University of Rajasthan Jaipur Rajasthan 302004 India l.p_riya@yahoo.co.in; c Department of Chemistry, Vivekananda Global University Jaipur Rajasthan India; d Department of Chemistry, Jai Narayan Vyas University Jodhpur Rajasthan India; e Department of Chemistry, IIS deemed to be University Jaipur Rajasthan India

## Abstract

The kinetics between paracetamol and *N*-chloro-*p*-toluenesulfonamide (chloramine-T) in the presence of an osmium(viii) catalyst in an alkaline medium was studied. The reaction followed second-order kinetics, and the effect of the catalyst indicated that an uncatalyzed reaction occurred simultaneously. The rate was slowed down by hydroxide ions. The oxidation product was spectrally confirmed to be quinone oxime in stoichiometry, where two moles of oxidant were required for each mole of the substrate. The thermodynamic quantities were also computed using the Eyring equation. A plausible reaction mechanism is suggested, accounting for all the experimental observations. To further support our proposed mechanism, density functional theory (DFT) computations at the M06-2X/6-31G* and b3lyp/lanl2dz/6-311*g (d,p) levels further confirmed the reaction mechanism that has been hypothesized based on the kinetic observations. The suggested mechanism is strongly supported by the computational results, which demonstrate a significant correlation between the activation energy barriers and the reactivity trends shown in the kinetic studies.

## Abstract 2

The reaction of paracetamol with chloramine-T in the presence of a transition metal catalyst reveals mechanistic pathways and rate dependencies, while oxidant speciation and Os(viii) reactivity in alkaline medium can be modeled mathematically.

## Graphical abstract

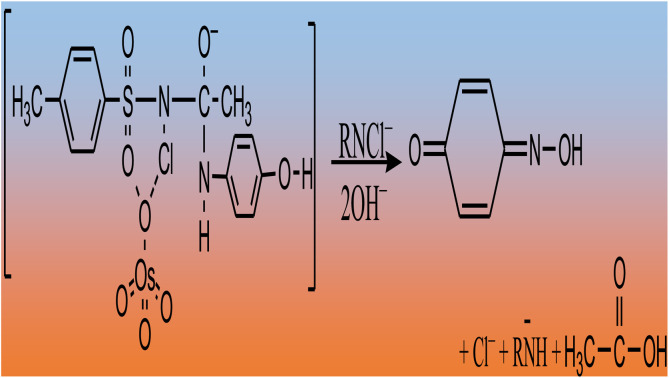

## Introduction

Chemical kinetics plays a crucial role in understanding and quantifying the rates of chemical reactions and the factors that influence them. It involves studying the reaction rates, the effects of different variables, the concentrations of reactants and products, the rearrangement of atoms, the formation of intermediates, the reaction mechanism, and the activation energy of the reaction. In chemical and pharmaceutical analyses, kinetics and thermal methods have gained significant interest. Kinetic methods, in particular, are highly valuable for drug analyses due to their ability to provide sensitive determinations using simple instrumental techniques. Here are a few reasons why kinetics is a preferred option for drug analyses: reaction rate determination, sensitivity, mechanism elucidation, variable effects, and activation energy determination. Overall, kinetic methods offer valuable tools to analytical chemists in drug analyses. They provide sensitive determinations, help in elucidating reaction mechanisms, and allow for the optimization of reaction conditions. By employing these methods, researchers can assess the quality, stability, and efficacy of pharmaceutical products, contributing to the development of safe and effective medications.

The continuous increase in the use of drugs and fertilizers has led to water pollution as various contaminants from industries such as pharmaceuticals and textiles are disposed into water bodies. Improper disposal of non-used medicines by individuals further exacerbates the problem. Pharmaceutical waste that enters water bodies can undergo chemical transformations into toxic substances that have detrimental effects on both human health and aquatic life. Paracetamol is a popular drug also known as *N*-(4-hydroxyphenyl)acetamide, which has wider applications in the pharmaceutical industry. It is also considered to be an antipyretic and analgesic compound with extremely useful therapeutic values in medicinal chemistry. As a commonly used analgesic and antipyretic drug, paracetamol has been detected in sewage treatment plant effluents at concentrations up to 6.0 μg l^−1^. This drug has not yet been exploited kinetically^1–3^ as far as its oxidation chemistry is concerned.

On the other hand, chloramine-T is widely utilized in medical, dental, food processing, and agricultural sectors due to its antimicrobial properties. It is commonly employed as a disinfectant and plays a vital role in the chlorination of drinking water during water treatment processes. Chloramine-T acts as an oxidizing agent and has the potential to oxidize paracetamol (acetaminophen) under specific conditions in both acidic^4–11^ and alkaline^12–17^ media. However, the reactions of this reagent are considerably slow in an alkaline medium. Therefore, the reactions of chloramine-T have been studied in the presence of various catalysts such as ruthenium(iii),^18^ palladium(ii)^19,20^ and osmium(viii)^21,22^ in an alkaline medium. Additionally, the elements which can easily form cations and have partially filled d-orbitals are reported as efficient catalysts in various organic oxidation reactions such as Os(viii), Ru(iii), Ir(iii), and Ag(i).^23^ The reactions catalyzed by osmium(viii) are interesting as the catalytic role of osmium(viii) is reported in various ways.^24–27^ Interestingly, chloramine-T species in an alkaline medium are not as well defined as those in an acidic medium. It is expected that the role of chloramine-T in such a reaction will be delineated in a logical manner. The use of catalysts alters the rates of kinetic reactions that are important in manufacturing and biochemical fields. In the absence of catalysts, the reaction is sluggish, but catalysts help to increase the oxidation process and the present catalyzed reaction is 4.3 times faster than the uncatalyzed reaction.

The scantiness of such oxidation studies^28,29^ has prompted us to analyze the oxidation kinetics of paracetamol (heretofore written as PCM) with chloramine-T to know more about its pattern of reactivity in an aqueous alkaline medium from the following viewpoints: first, the chloramine-T species speciation in the alkaline medium can be achieved, and second, the pattern of reactivity of osmium(viii) as a catalyst can also be understood for a proper proposition of the reaction mechanism. Oxidation reactions are useful in the synthesis process of pharmaceutical drugs and organic compounds. Redox reactions contribute to the modification of existing groups in the compound. Paracetamol is a precursor of many pharmaceutical drugs and dyes. This study can be useful to determine paracetamol in drugs and its degradation. Therefore, understanding the role of mechanism of decomposition of this drug in an aqueous medium is required to clear the probable pathway of its oxidation.

The specific products formed as a result of the reaction between paracetamol and chloramine-T can vary depending on factors such as the concentration of chloramine-T, pH, temperature, and reaction time. One product that can be formed is quinone oxime, along with its salts. These products are considered as synthons, which are building blocks for the synthesis of new biologically active derivatives. They find wide-ranging applications in the field of applied biological sciences and serve as bioactive agents. Understanding the potential reaction between paracetamol and chloramine-T is important due to its implications for water treatment processes and environmental impact. The formation of potentially bioactive compounds from this reaction highlights the need for effective water treatment strategies to mitigate the presence of pharmaceutical contaminants in water systems. Additionally, the study of the reaction conditions and products formed contributes to the broader understanding of the chemical transformations undergone by pharmaceuticals in the environment, aiding in the development of strategies to minimize their impact on human health and aquatic life.

Further, the present work has practical importance in both pharmacy and medicine due to the following facts: drug stability: paracetamol (acetaminophen) is a widely used analgesic and antipyretic drug. Understanding its stability in various environments is crucial for determining its shelf life, safe storage conditions, and its behavior under different physiological conditions. This study provides insights into the oxidative degradation of paracetamol. Knowing the degradation products is important because some degradation products can be toxic or may lead to reduced efficacy of the drug. The use of osmium(viii) as a catalyst in oxidative processes highlights the role of metal catalysts in pharmaceutical manufacturing, where catalysts are often used to accelerate reactions, enhance yields, and achieve selective transformations in drug synthesis. Catalysis can lead to more efficient processes, reducing the amount of waste and energy required for drug production. This is particularly important in large-scale pharmaceutical manufacturing. The study's computational approach helps in predicting the stability and reactivity of paracetamol and similar drugs. This can be applied in drug design to predict the behaviour of new compounds, allowing for the optimization of their stability and efficacy before clinical trials. Understanding the mechanistic pathways of drug degradation can inform the design of more stable analogues of paracetamol or new drugs that resist degradation under certain conditions. By studying the degradation products, researchers can assess the safety profile of both the parent drug and its degradation products. This is important for ensuring that the drug remains safe and effective throughout its intended use. Regulatory agencies require detailed information on drug stability and degradation pathways. This study contributes to the data needed for regulatory submissions, particularly for the approval of generic versions of paracetamol or new formulations. Pharmaceutical companies can use the findings to develop quality control methods to detect degradation products, ensuring that only stable and effective drug products reach the market. This work also has broader implications for understanding oxidative stress and how drugs like paracetamol may interact with oxidative agents in the body, which can be relevant for understanding drug interactions and side effects.

Hence, this study is significant for both pharmacy and medicine as it provides valuable insights into the stability, degradation, and safety of paracetamol. It also emphasizes the importance of catalytic processes and computational methods in drug development, manufacturing, and quality control.

## Experimental

### Materials and methods

*N*-Chloro-*p*-toluenesulfonamide was taken as its sodium salt (E. Merck) and its solution was prepared in double-distilled water. The aqueous solution is quite stable if it is contained in glass vessels painted black from the outside, and the stability is enhanced as the decomposition is eliminated due to diffused photo-light. The solution of chloramine-T was standardized iodometrically^30,31^ by titrating an aliquot of this solution in the presence of KI in an acidic medium. The liberated iodine was titrated against a hypo solution using starch as an indicator with a sharp end point. Paracetamol (Acros) was used as received without any purification. An aqueous solution of paracetamol was also kept in brown-colored glass bottles in a refrigerator at a temperature of ∼5 °C. However, a fresh solution of paracetamol was always employed. Osmium(viii) was employed as received as osmium tetroxide (Johnson Matthey). Its solution was prepared in 0.5 M sodium hydroxide and the solution was standardized iodometrically. The solution of osmium(viii) was kept in dark glass bottles to avoid photochemical decomposition. Such a stock solution of osmium(viii) was kept in a refrigerator at ∼5 °C.

### Kinetic procedure

The reaction mixtures containing paracetamol, sodium hydroxide and other ingredients except chloramine-T were taken in glass-stoppered Erlenmeyer flasks, which were immersed in a thermostated water bath at 35 °C ± 0.1 °C unless specified otherwise. The temperature pre-equilibrated solution of chloramine-T was added into the reaction mixture to initiate the reaction; the time of initiation was recorded when half of the solution from the pipette was released into the reaction mixture. The reaction mixture was vigorously shaken and an aliquot (5 or 10 cm^3^) was taken out at different time intervals and then discharged into a 10% KI solution. The free I_2_ was titrated against a hypo solution using starch as an indicator. The reaction was studied under pseudo-first-order conditions ([PCM]) ≫ 10 × ([CAT]). The second-order rate constants (dm^3^ mol^−1^ s^−1^) calculated from second-order plots and those from first-order plots were in agreement (Table 1). Furthermore, the coefficient of variation (CV) values range from 0.65% to 1.47%, indicating very low relative variability among the triplicate measurements, which suggests good reproducibility. The intraclass correlation coefficient (ICC) value of 0.999, which is very close to 1.0, also indicates an excellent level of agreement and consistency among the triplicate measurements. Therefore, the measurement of concentration over time for this oxidation reaction is highly reliable and reproducible.

**Table 1:** Pseudo-first-order rate constants (*k*′) s^−1^ and second-order rate constants (*k*′′) in the reaction of paracetamol and chloramine-T in an aqueous alkaline medium

[NaOH] = 0.05 mol l^−1^ and 35 °C			
10^2^ [PCM] mol l^−1^	10^3^ [CAT] mol l^−1^	10^4^ (*k*′) s^−1^	10^3^ (*k*′′) l mol^−1^ s^−1^
4.0	1.0	1.91	4.78 (4.77)
4.0	1.5	1.91	4.77 (4.77)
4.0	2.5	1.91	4.76 (4.77)
4.0	3.0	1.91	4.77 (4.77)
4.0	3.5	1.91	4.78 (4.77)
4.0	4.0	1.91	4.77 (4.77)
4.0	4.5	1.91	4.77 (4.77)
4.0	5.0	1.91	4.71 (4.77)
5.0	1.0	2.303	4.72 (4.66)
5.0	1.5	2.303	4.71 (4.66)
5.0	2.5	2.303	4.74 (4.66)
5.0	3.0	2.303	4.72 (4.66)
5.0	3.5	2.303	4.71 (4.66)
5.0	4.0	2.303	4.74 (4.66)
5.0	4.5	2.303	4.69 (4.66)
5.0	5.0	2.303	4.71 (4.66)
1.0	2.0	0.47	4.75 (4.70)
2.0	2.0	0.96	4.73 (4.79)
2.5	2.0	1.15	4.76 (4.60)
3.0	2.0	1.44	4.72 (4.79)
3.5	2.0	1.72	4.71 (4.90)
4.0	2.0	1.91	4.73 (4.77)
4.5	2.0	2.11	4.75 (4.68)
5.0	2.0	2.303	4.72 (4.60)
1.0	3.0	0.47	4.76 (4.70)
2.0	3.0	0.96	4.74 (4.70)
2.5	3.0	1.15	4.75 (4.79)
3.0	3.0	1.44	4.71 (4.60)
3.5	3.0	1.72	4.73 (4.90)
4.0	3.0	1.91	4.74 (4.77)
4.5	3.0	2.11	4.76 (4.68)
5.0	3.0	2.303	4.72 (4.60)

Figures in the parenthesis are calculated second-order rate constants.

### Statistical data analysis

The pseudo-first-order rate constants were determined from the absorbance-time data using the StatGraphics Centurion Software (19 × 64). Correlation analysis was performed using Microsoft Excel (LINEST function). The quality of fit was assessed using the correlation coefficient (*R*^2^) for linear regression and the standard deviation (SD).

### Stoichiometry and product analysis

The stoichiometry of the reaction was calculated by taking an excess concentration of chloramine-T over that of paracetamol. The excess chloramine-T was estimated iodometrically, which corresponds to the stoichiometry of the reaction, as represented by eqn (1):1
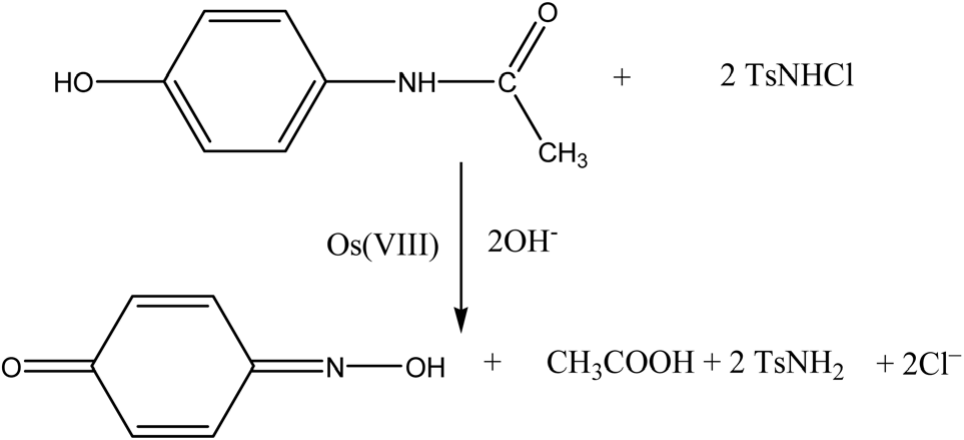


Product analysis was carried out by allowing the reaction mixture to stand for about 24 hours in the presence of an excess of either [CAT] or [PCM]. The oxidation product of paracetamol by chloramine-T in a basic medium was identified as quinone oxime by chromatography, IR spectroscopy and NMR spectroscopy. This product oxime has also been established earlier.

Quinone oxime was purified from other reaction impurities by column chromatography. TLC were conducted on Merck-Silica gel G plates in dichloromethane:methanol (9 : 1, v/v) and in column, chromatographic fractionation silica gel (60–120 mesh) was used and iodine was used as the developing reagent. This product was further characterized by spectral techniques.

### Spectral analysis

In the IR spectrum, an absorption band at 1652 cm^−1^ is due to >C=O stretching, the band at 1615 cm^−1^ is due to C=N stretching and the band at 3332 cm^−1^ is due to O–H stretching.

In ^1^H NMR spectrum, a doublet is obtained at d 6.69 ppm due to aromatic two protons and another doublet at d 6.49 ppm is due to another type of two aromatic protons and a singlet at d 1.999 ppm due to the –OH group.

**Fig. 1:**
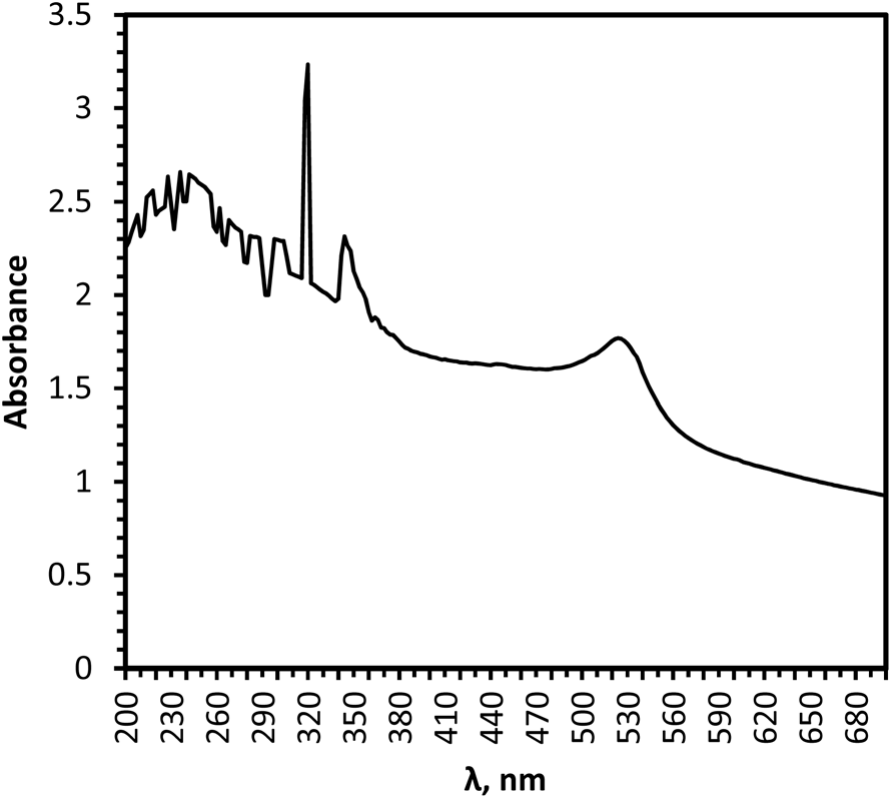
UV spectrum of quinone oxime.

The product was also established by UV-visible spectrophotometry to be quinone oxime, as it absorbs maximum at *λ*_max_ 318 nm (Fig. 1). The *λ*_max_ value reported^32^ for quinone oxime is 320 nm. Since quinone absorbs at 410 nm, the product is not quinone.^33^

### Spectrophotometric results

The reagents such as chloramine-T and paracetamol were taken separately and *λ*_max_ was determined to be 220 nm and 240 nm, respectively, as both are transparent in the visible region. The mixture of chloramine-T and paracetamol shows *λ*_max_ at 225 nm, confirming that there is no complex formation between chloramine-T and paracetamol. Further, there was no change in broad *λ*_max_ of osmium(viii) upon the addition of paracetamol ruling out any possibility of complex formation between the catalyst and paracetamol. These observations were in agreement with those obtained kinetically, as no evidence was obtained for complexation between paracetamol and osmium(viii).

## Results and discussion

**Fig. 2:**
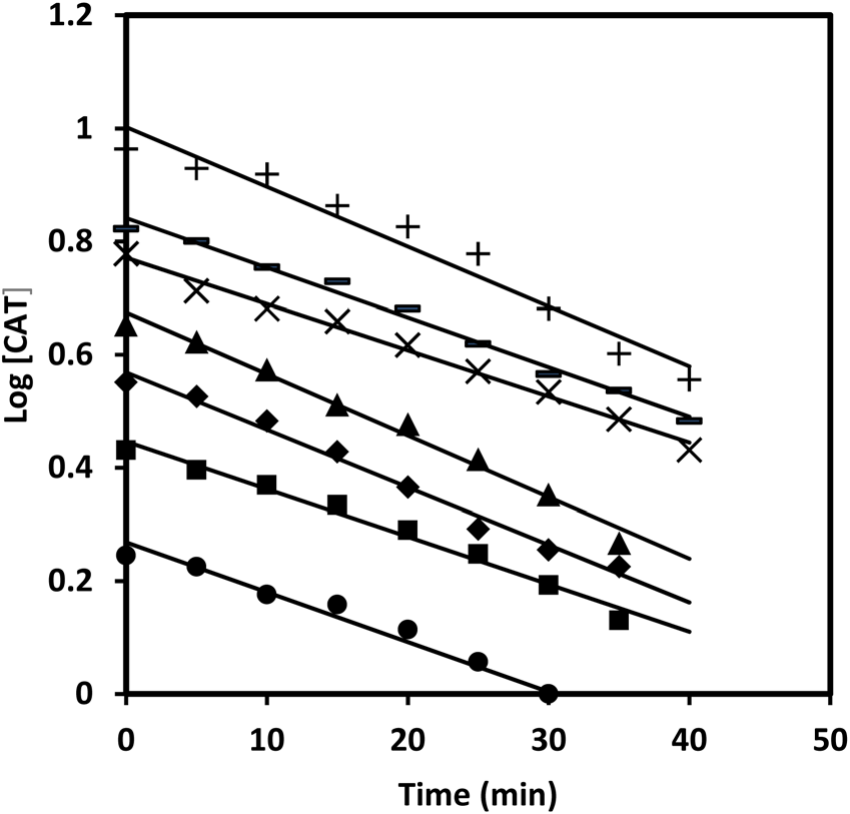
Pseudo-first-order plots of chloramine-T [PCM] = 5.0 × 10^−2^ mol l^−1^; [NaOH] = 0.05 mol l^−1^; [Os(viii)] = 5 × 10^−5^ mol l^−1^ [CAT] = (1.0 ●; 1.5 ■; 2.0 ◆; 2.5 ▲; ×3.0; <svg xmlns="http://www.w3.org/2000/svg" version="1.0" width="32.333333pt" height="16.000000pt" viewBox="0 0 32.333333 16.000000" preserveAspectRatio="xMidYMid meet"><metadata> Created by potrace 1.16, written by Peter Selinger 2001-2019 </metadata><g transform="translate(1.000000,15.000000) scale(0.014583,-0.014583)" fill="currentColor" stroke="none"><path d="M80 480 l0 -160 960 0 960 0 0 160 0 160 -960 0 -960 0 0 -160z"/></g></svg> 4.0; +5.0) × 10^−3^ mol l^−1^ and 35 °C.

The effect of chloramine-T was studied by varying the concentration in pseudo-first-order conditions keeping other reaction ingredients constant at 35 °C. Pseudo-first-order plots were made and the pseudo-first-order rate constants (*k*′, s^−1^) were evaluated, which were found to be independent of initial gross concentrations of chloramine-T, confirming first-order dependence with respect to chloramine-T. Typical pseudo-first-order plots (*R*^2^ = 0.9595 to 0.9914) are given in Fig. 2.

**Fig. 3:**
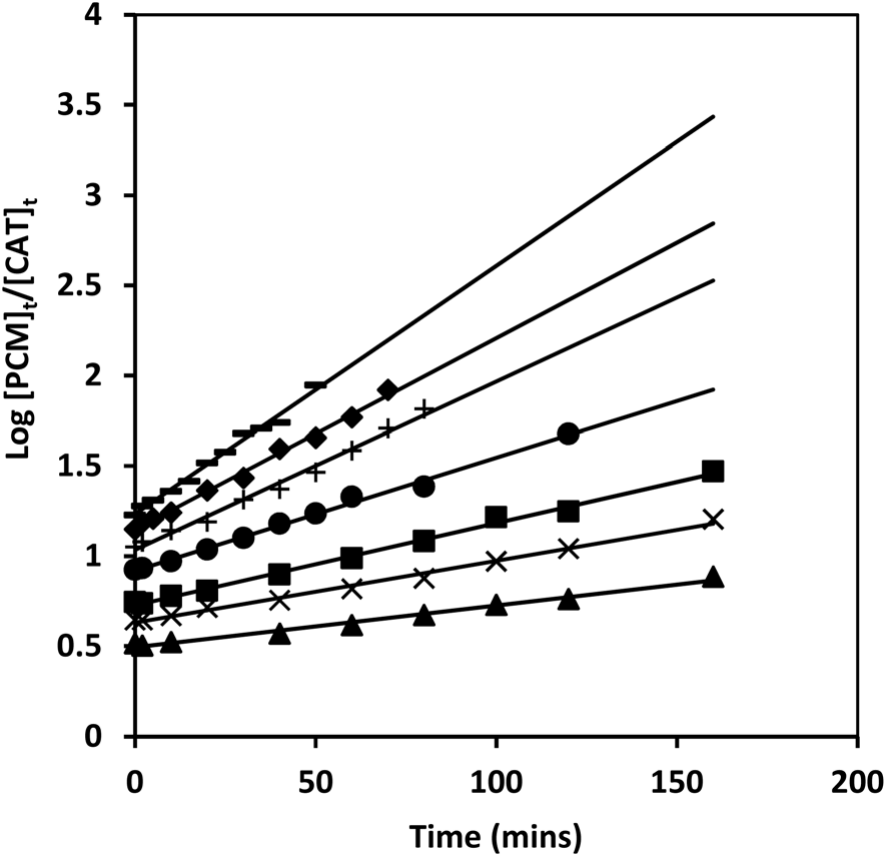
Second-order plots of paracetamol [CAT] = 2.0 × 10^−3^ mol l^−1^; [NaOH] = 0.05 mol l^−1^; [Os(viii)] = 5.0 × 10^−5^ mol l^−1^ and 35 °C; [PCM] = (▲ 1.0; ×2.0; ■ 2.5; ●3.5; +4.0; ◆ 4.5; −5.0) × 10^−2^ mol l^−1^.

The effect of paracetamol was studied by varying the concentration under pseudo-first-order conditions keeping other reaction ingredients constant at 35 °C. The pseudo-first-order rate constants (*k*′, s^−1^) were evaluated and a plot of rate constants against the concentration of paracetamol yielded a straight line passing through the origin, indicating first-order dependence with respect to the substrate. Second-order plots (*R*^2^ = range 0.9877 to 0.9952) were also made wherever the concentration of reactants is comparable (Fig. 3).

The variation in osmium(viii) from 2.0 × 10^−5^ mol l^−1^ to 8.0 × 10^−5^ mol l^−1^ is probed keeping other reaction ingredients constant at 35 °C. This showed the first order dependence (*R*^2^ = 1.0) with respect to the catalyst.

**Fig. 4:**
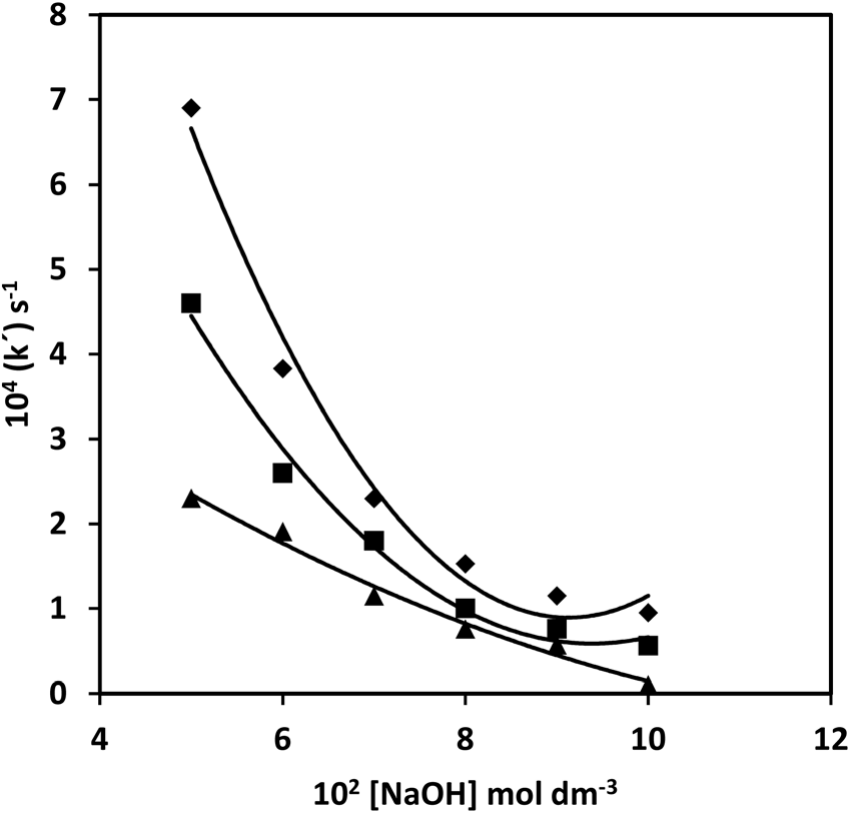
Effect of hydroxide ion concentration [CAT] = 2.0 × 10^−3^ mol l^−1^; [PCM] = 5.0 × 10^−2^ mol l^−1^; [Os(viii)] = 5.0 × 10^−5^ mol l^−1^, Temp. = ▲ 35; ■ 40 and ◆ 45 °C.

The hydroxide ion concentration was varied from 5.0 × 10^−2^ mol l^−1^ to 10.0 × 10^−2^ mol l^−1^ keeping other reaction ingredients constant at 35 °C, 40 °C, and 45 °C. The rate decreases with the increase in hydroxide ion concentration in a complex manner, as shown in Fig. 4.

The ionic strength effect was also investigated by using sodium nitrate fixing the other reaction ingredients constant. The rate was independent of the changing ionic strength.

The reactions were also occurred in a thermostated water-bath and acryl nitrile/acrylic acid was added during the progress of the reaction. There was no white sediment in the reaction mixture even after a long time of 24 hours. This shows that no free radical participates in the reaction. It appears that the radical reacts in a solvent cage and does not defuse out of it under such experimental conditions. Had it not been a situation, a white precipitate would have been obtained in the reaction mixture on addition of acryl nitrile/acrylic acid after polymerizing the monomer.

The reaction was also examined at different temperatures, namely, 30 °C, 35 °C, 40 °C, 45 °C and 50 °C keeping other reaction ingredients constant. A straight line was found by constructing the Eyring plot^34^ between ln(*k*′/*T*) and 1/*T*. The large (−)ve entropy shows a sufficiently stabilized transition state.

## Mechanism of oxidation

The sodium salt of chloramine-T is a strong electrolyte reported in different forms, which are governed by equilibria (2)–(6):^1,2,35–37^

2 *p*-CH_3_-C_6_H_4_SO_2_NClNa ⇌ *p*-CH_3_-C_6_H_4_SO_2_NCl^−^ + Na^+^

3 *p*-CH_3_-C_6_H_4_SO_2_NCl^−^ + H^+^ ⇌ *p*-CH_3_C_6_H_4_NHCl

4 *p*-CH_3_-C_6_H_4_SO_2_NHCl + H_2_O ⇌ *p*-CH_3_C_6_H_4_SO_2_NH_2_ + HOCl

5 2 *p*-CH_3_-C_6_H_4_SO_2_NHCl ⇌ *p*-CH_3_C_6_H_4_SO_2_NH_2_ + *p*-CH_3_C_6_H_4_NCl_2_

Hypochlorous acid ionizes, as shown in eqn (6):

6 HOCl H^+^ + OCl^−^

In an acidic medium, the reactions of chloramine-T are known to be catalyzed by chlorine ions through an intermediate species such as *p*-CH_3_-C_6_H_4_SO_2_NCl^*d*+^-Cl^*d*−^, which on redox rupturing yields Cl_2_. Thus, it is the chlorine that is responsible for chloride ion catalysis.

In view of these observations, the reactive chloramine-T species are pH dependent, which in the acidic medium are *p*-CH_3_-C_5_H_4_SO_2_NHCl, HOCl, and *p*-CH_3_-C_6_H_4_SO_2_NCl_2_ and in the alkaline medium are *p*-CH_3_-C_6_H_4_SO_2_NCl^−^ and OCl^−^. OCl^−^ species is only effective in base-catalyzed reactions. Moreover, the rate of the reaction should be slowed down by *p*-CH_3_-C_6_H_4_SO_2_NH_2_, which is the reduced product of chloramine-T in the acidic medium. Since the title reaction is inhibited by hydroxide ions and the rate of the reaction is not affected by PTS, the participation of OCl^−^ species can be ruled out conveniently. Moreover, *p*-CH_3_-C_6_H_4_NHCl_2_, *p*-CH_3_C_6_H_4_NCl_2_ and HOCl are reactive species only in an acidic medium, and as such, these are also ruled out in the title reaction. The reaction is not catalyzed by chloride ions. Had it been catalyzed by chloride ion, *p*-CH_3_C_6_H_4_SO_2_NHCl would have been an effective species. Thus *p*-CH_3_-C_6_H_4_SO_2_NHCl to be the reactive form is also ruled out. Thus, in an alkaline medium, the reactive form of chloramine-T is *p*-CH_3_C_6_H_4_SO_2_NCl^−^ (RNCl^−^, where R = *p*-CH_3_C_6_H_4_SO_2_) based on these discussions.

Further if calculations based on Bishop and Jennings^38^ are considered to be any guide, the RNCl^−^ concentration is remarkably higher than that of RNHCl in an alkaline medium.

Osmium(viii) in an alkaline medium is reddish brown in color, in which [OsO_4_(OH)_2_^2−^] is reported^39^ to be the predominant species of the catalyst. Such a species is converted into [OsO_3_(OH)_3_^−^] species in a dilute alkaline medium. Since the rate decreases with the increasing concentration of hydroxide ions, [OsO_3_(OH^−^)_3_] appears to be the reactive form of osmium(viii).

Thus, considering RNCl^−^ as the reactive species of chloramine-T and [OsO_3_(OH)_3_^−^] as the reactive form of the catalyst, the following reaction mechanism can be envisaged to account for the experimental observations.
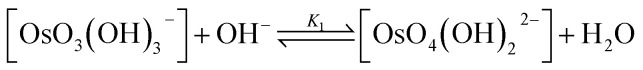

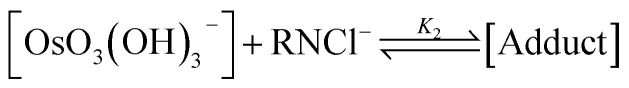

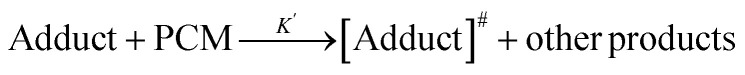

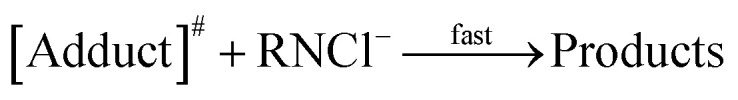


The loss of chloramine-T leads to the rate law (7):7
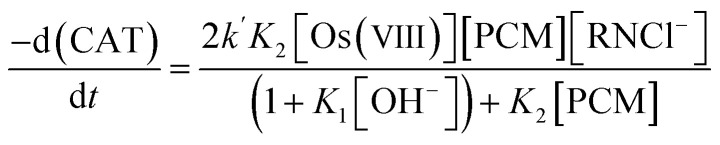


Since the order with respect to paracetamol is one, the inequality (1 + *K*_1_[OH^−^]) ≫ *K*_2_[PCM] is a valid assumption. This reduces the rate law (7)–(9):8
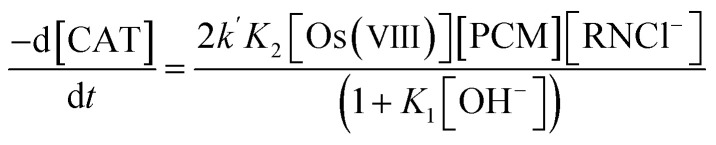
where [Os(viii)], [PCM] and [RNCl^−^] are the gross analytical concentrations of the catalyst, substrate and the oxidant, respectively.or9
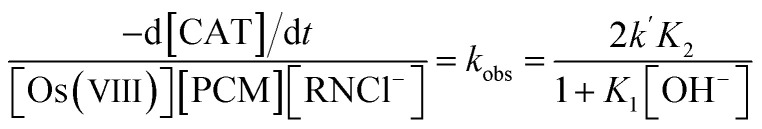
where *k*_obs_ is an observed third-order rate constant. Since *K*_2_ is an insignificant equilibrium constant, this further reduces the rate law (9) to (10):10
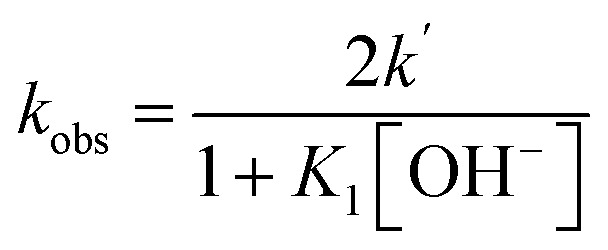


**Fig. 5:**
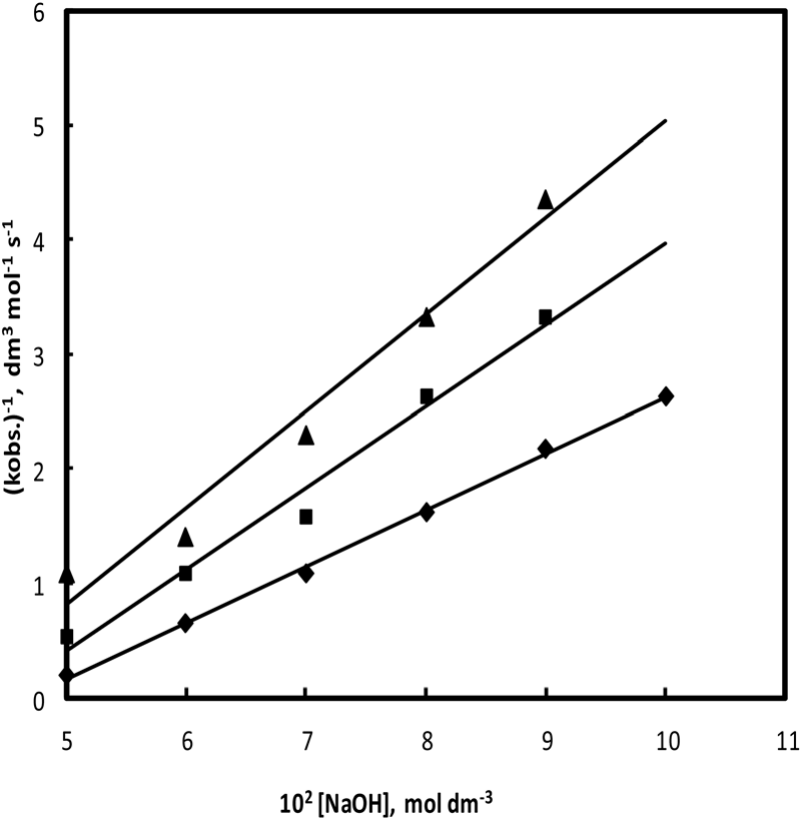
Plot of (*k*_obs_)^−1^*versus* [OH^−^] [CAT] = 2.0 × 10^−3^ mol l^−1^; [PCM] = 5.0 × 10^−2^ mol l^−1^; [Os(viii)] = 5.0 × 10^−5^ mol l^−1^; Temp. = ◆ 45, ■ 40, ▲ 35 °C.

Straight lines were found by plotting between (*k*_obs_)^−1^ and [OH^−^] from the double reciprocal of eqn (10), with a non-zero intercept (Fig. 5). From the intercept, *k*′ was evaluated to be 0.65, 0.5 and 0.35 dm mol^−1^ at 35 °C, 40 °C and 45 °C, respectively. *K*_1_ was obtained from the ratio of intercept and slope, which was found to be 0.72, 0.63 mol l^−1^.

The properties and mechanism of paracetamol drug were distinguished from the molecular properties of its analogues. The structure and binding of this drug are known to play an important role in its pharmacological actions. Among its analogues, paracetamol is an important one and applied as an antipyretic and analgesic drug. The substituents at the para position of the aromatic ring, along with the lone pair on the nitrogen of the NHCOCH_5_ group capable of non-covalent interactions with osmium, play a significant role in influencing variations in transition-state properties such as pharmacological activity, reduction potential, and overall stability*.*

## Theoretical calculations and mechanistic validation

Theoretical investigations were carried out using Density Functional Theory (DFT) to support the proposed reaction mechanism and evaluate thermodynamic consistency with experimental results. Preliminary calculations were performed using the Spartan software,^40^ employing the M06-2X functional with the 6-31G* basis set, an approach shown to be well suited for thermal and kinetic predictions.^41^ These computations revealed activation energy barriers consistent with the experimental reactivity trends observed in kinetic studies, particularly for the quinone-oxime system and under polarizable continuum model (PCM) conditions.

**Fig. 6:**
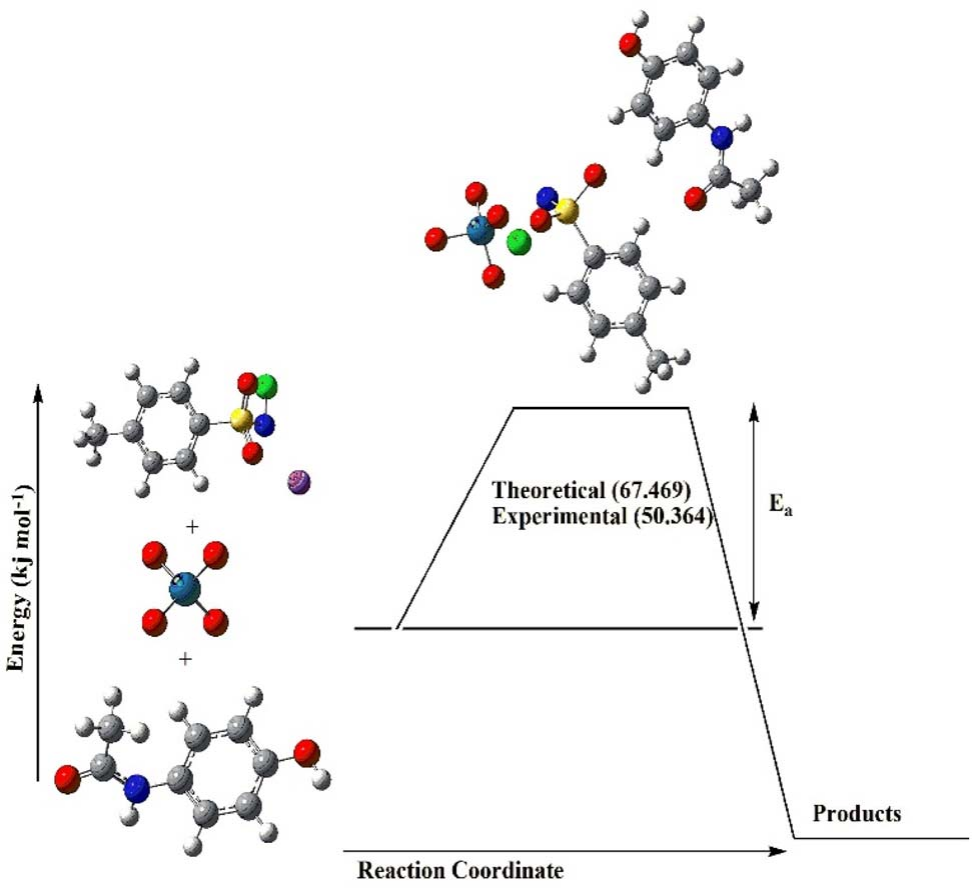
Reaction coordinates for the rate-determining step. Acetanilide ≫ Paracetamol > 2-hydroxyacetanilide > 3-hydroxyacetanilide (metacetamol) > 4-ethoxyacetanilide (phenacetin).

To gain deeper insights into the reaction pathway, further DFT calculations were conducted using the Gaussian software. All stationary geometries as reactants, intermediates, products, and transition states were optimized using the hybrid B3LYP functional with a mixed LANL2DZ basis set for heavier atoms and 6-311G*(d,p) for lighter elements. Solvent effects were accounted for using the Polarizable Continuum Model (PCM) with integral equation formalism (IEFPCM), simulating aqueous conditions. Frequency calculations were performed to validate the nature of each stationary point. All equilibrium geometries were confirmed by the absence of imaginary frequencies, whereas the transition state exhibited a single imaginary frequency, confirming its identity as a first-order saddle point. As shown in Fig. 6, the calculated activation energy for the rate-determining step was 67.469 kJ mol^−1^, closely aligning with the experimentally derived activation energy of 50.36 kJ mol^−1^ obtained through kinetic modelling (from observed rate constants, *k*′). Table 2 presents the optimized structural parameters, and Table 3 summarizes the key thermodynamic properties. The good agreement between theoretical and experimental activation energies reinforces the proposed mechanistic pathway and underscores the reliability of the computational approach.

**Table 2:** Structural parameters and optimized structures of paracetamol and quinone oxime calculated by the DFT M06-2X/6-31G* method

Parameters	Paracetamol	Quinone-oxime	
Molecular structure	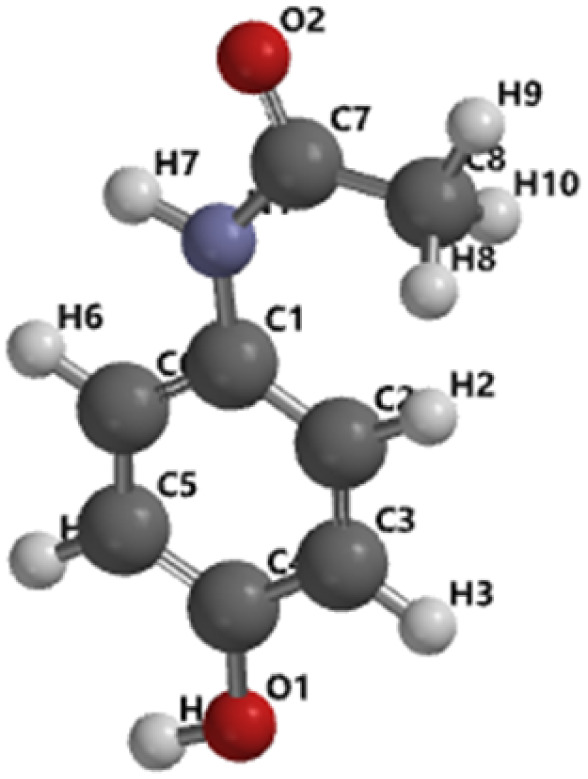	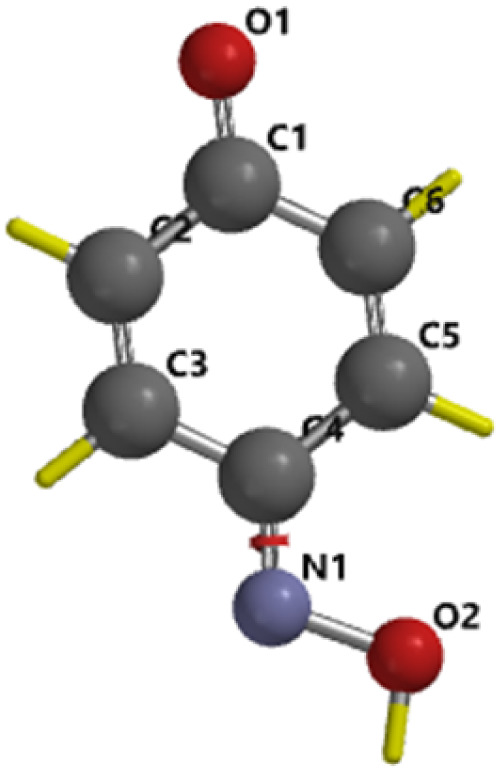	
Energy	423.69 kJ mol^−1^	271.67 kJ mol^−1^	
C_4_–O_1_	1.362 A°	C_1_–O_1_	1.474 A°
C_1_–C_2_	1.394 A°	C_1_–C_2_	1.336 A°
C_2_–C_3_	1.401 A°	C_2_–C_3_	1.485 A°
C_3_–C_4_	1.387 A°	C_3_–C_4_	1.488 A°
C_4_–C_5_	1.386 A°	C_4_–C_5_	1.337 A°
C_5_–C_6_	1.399 A°	C_5_–C_6_	1.474 A°
C_1_–C_6_	1.406 A°	C_1_–C_6_	1.224 A°
C_1_–N_1_	1.406 A°	C_4_–N_1_	1.299 A°
C7–N1	1.361 A°	N_1_–O_1_	1.400 A°
C_7_–C_8_	1.498 A°	—	—
C_7_–O_2_	1.235 A°	—	—
*θ* (C_3_–C_4_–O_1_)	117.92°	*θ* (O_1_–C_1_–C_6_)	121.36°
*θ* (C_3_–C_4_–C_5_)	120.41°	*θ* (C_1_–C_6_–C_5_)	121.54°
*θ* (C_2_–C_1_–C_6_)	118.21°	*θ* (C_1_–C_2_–C_3_)	121.31°
*θ* (C_2_–C_1_–N_1_)	125.97°	*θ* (C_3_–C_4_–C_5_)	115.97°
*θ* (C_1_–N_1_–C_7_)	134.80°	*θ* (C_5_–C_4_–N_1_)	126.22°
*θ* (N_1_–C_7_–O_2_)	117.7°	*θ* (C_3_–C_4_–N_1_)	117.81°
*θ* (O_2_–C_7_–C_8_)	120.08°	*θ* (C_4_–N_1_–O_2_)	112.11°
*Δ* (Dihedral angle)	Almost same (180°)		

**Table 3:** Experimental thermodynamic parameters calculated using the DFT M06-2X/6-31G* method*

Δ*H*^#^, kJ mol^−1^	Δ*S*^#^, J K^−1^ mol^−1^	*E* ^#^_a_, kJ mol^−1^	Δ*G*^#^, kJ mol^−1^	Ln *A*
87.18 ± 3.0	−31.94 ± 0.9 × 10^1^	89.78 ± 3.0	97.02	26.66 ± 1.14
89.91*	−35.33*	92.47*	104.21*	

**Scheme 1:**
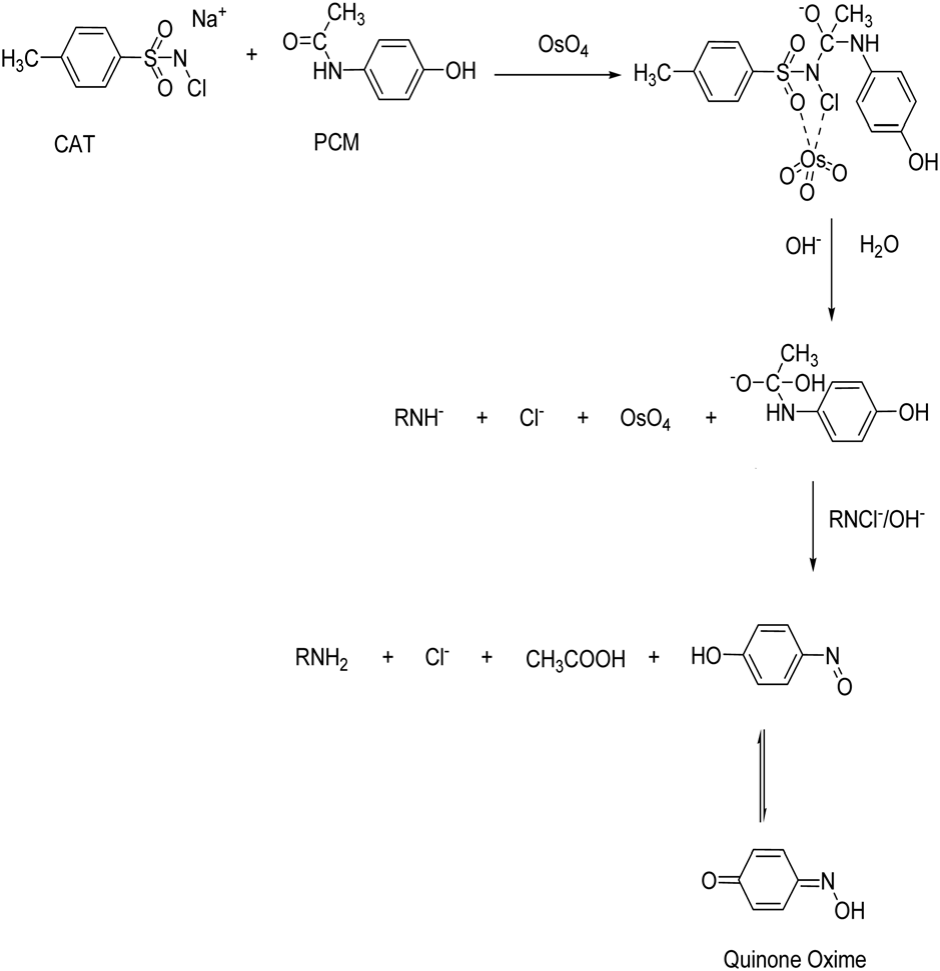
Reaction mechanism of the Os(viii)-catalysed reaction between PCM and CAT.

The catalysis of paracetamol analogues (acetanilide, 2-hydroxyacetanilide), 3-hydroxyacetanilide (metacetamol) and 4-ethoxyacetanilide (phenacetin) was studied by author. Spectral changes confirmed that the product formed in this catalyzed reaction is oxime. A variation in the rate for different analogues may be accounted for a change in the strength (−I, +I, −R, +R) of these compounds, causing changes in the availability of the lone pair of nitrogen in amido groups. A similar degradative process is involved for paracetamol analogues with *Penicillium* species, as also reported previously.^42^ This result reveals that the reaction proceeds *via* a common mechanism. The order of reactivity is as follows:

Thus far, the mode of electron transfer from paracetamol to *N*-chloro-*p*-toluenesulfonamide catalyzed by osmium(viii) can be tentatively understood from the following Scheme 1 of reaction events. A molecular complex of these reagents is formed, which most likely corresponds to the structure shown in Scheme 1.

The electron density around the nitrogen atom in *N*-chloro-*p* toluenesulfonamide decreases, which weakens the N–Cl bond. The hydride ion abstracting capacity due to the subsequent electrophilic characteristic of *N*-chloro-*p*-toluenesulfonamide increases and results in an interaction with paracetamol. Such a cyclic structure appears to be a mode of electron transfer in the reaction. This is not unique, as such a complex has also been suggested earlier^43^ in osmium(viii)-catalyzed oxidation of carboxylic acid by *N*-chloro-*p*-toluenesulfonamide.

## Conclusions

The study of the kinetics and mechanism of oxidation of paracetamol by chloramine-T in an aqueous alkaline medium provides valuable insights into the reaction's behavior and offers potential practical applications: (1) the study on the oxidation of paracetamol can help understand its stability and degradation under different conditions, especially in the presence of reactive species like chloramine-T. This is crucial for pharmaceutical formulations, where maintaining the stability of drugs is important for efficacy and safety. (2) By knowing how paracetamol decomposes under such conditions, manufacturers can refine the preservation techniques to prolong the shelf life of paracetamol-based products. (3) The mechanism of oxidation reactions involving chloramine-T provides a deeper understanding of redox processes in aqueous solutions, especially under alkaline conditions. This is valuable in industrial processes that involve oxidizing agents, such as water disinfection, or in processes where controlled oxidation is needed such as chemical synthesis or waste treatment. (4) The study's findings on rate laws and reaction intermediates are essential for improving the efficiency and safety of processes using oxidizing agents in chemical industries. (5) Knowledge about the rate of oxidation and reaction intermediates can offer insights into the potential toxicity of the degradation products of paracetamol. For example, if dangerous by-products are formed during the reaction, this could have implications for both the environment and human health. (6) The study's kinetics can be applied to optimize industrial or laboratory-scale oxidation reactions. By knowing the precise concentration of chloramine-T, reaction temperature, and pH conditions, industries can achieve desired reactions more efficiently and cost-effectively. (7) The study's findings on the activation energy of the reaction provide insights into how temperature influences the rate of oxidation. In industrial applications, maintaining a consistent temperature is important to control reaction rates and avoid degradation of useful compounds.

In conclusion, the practical significance of the study lies in its application to pharmaceutical stability, environmental protection, and industrial chemical processes. The findings suggest that the reaction conditions such as pH, concentration of chloramine-T, and temperature need to be carefully controlled for desired outcomes. Moreover, the study emphasizes the need for more research studies on optimizing these conditions for practical applications in industries and environmental clean-up.

## Author contributions

The manuscript was written through contributions of all authors. All authors have given approval to the final version of the manuscript.

## Conflicts of interest

There are no conflicts to declare.

## Data Availability

All quantum chemical computations were performed using both Gaussian 09W and Spartan'20 software packages. Geometry optimizations and transition state searches were carried out using density functional theory (DFT). For Gaussian 09W,^44^ the B3LYP functional was used in combination with the 6-311G(d,p) basis set for main-group elements (H, C, N, O, S, and Cl), and the LANL2DZ effective core potential (ECP) and basis set were employed for osmium (Os). Additionally, Spartan'20 (ref. 40) was used to validate and visualize selected transition state geometries and intermediates using the M06-2X functional with the 6-31G basis set*, chosen for its computational efficiency and reliability in modeling thermochemical and kinetic properties of organometallic systems.
